# Supplementary material for: Clinical Trends in Management of Locally Advanced ESCC: Real-World Evidence from a Large Single-Center Cohort Study
Source: Cancers (Basel). 2022 Oct 10;14(19):4953. doi: 10.3390/cancers14194953 (PMC9564200; doi:10.3390/cancers14194953)
Supplement: Supplementary file 1 [file cancers-14-04953-s001.zip › cancers-1859609-supplementary.pdf]

Supplement Table. Stratified multivariate Cox proportional-hazard model by stage and type of treatment

Stage	Variables	Hazard Ratio [95% CI]	P-value
T1-2	Surgery only	Ref	
	Neoadjuvant CCRT with surgery	0.926 [0.635 - 1.352]	0.691
	Surgery with adjuvant chemotherapy	0.716 [0.496 - 1.033]	0.074
	Definitive CCRT	1.141 [0.628 - 2.073]	0.666
T3-4	Surgery only	Ref	
	Neoadjuvant CCRT with surgery	0.704 [0.572 - 0.866]	**< 0.001**
	Surgery with adjuvant chemotherapy	0.813 [0.632 - 1.047]	0.109
	Definitive CCRT	0.959 [0.732 - 1.255]	0.758
N0	Surgery only	Ref	
	Neoadjuvant CCRT with surgery	0.769 [0.472 - 1.255]	0.293
	Surgery with adjuvant chemotherapy	0.337 [0.083 - 1.373]	0.129
	Definitive CCRT	0.726 [0.362 - 1.455]	0.367
N1-3	Surgery only	Ref	
	Neoadjuvant CCRT with surgery	0.721 [0.594 - 0.875]	**< 0.001**
	Surgery with adjuvant chemotherapy	0.649 [0.525 - 0.802]	**< 0.001**
	Definitive CCRT	1.152 [0.886 - 1.497]	0.29
M0	Surgery only		
	Neoadjuvant CCRT with surgery	0.751 [0.622 - 0.907]	**0.003**
	Surgery with adjuvant chemotherapy	0.715 [0.576 - 0.888]	**0.002**
	Definitive CCRT	0.871 [0.653 - 1.162]	0.348
M1	Surgery only	Ref	
	Neoadjuvant CCRT with surgery	0.869 [0.476 - 1.586]	0.648
	Surgery with adjuvant chemotherapy	0.557 [0.276 - 1.126]	0.103
	Definitive CCRT	1.678 [1.026 - 2.743]	0.039
